# Simulating the Interplay between the Uptake of Inorganic Phosphate and the Cell Phosphate Metabolism under Phosphorus Feast and Famine Conditions in *Chlorella vulgaris*

**DOI:** 10.3390/cells10123571

**Published:** 2021-12-17

**Authors:** Tatiana Yu. Plyusnina, Sergei S. Khruschev, Polina V. Fursova, Alexei E. Solovchenko, Taras K. Antal, Galina Yu. Riznichenko, Andrei B. Rubin

**Affiliations:** 1 Faculty of Biology, Lomonosov Moscow State University, Leninskie Gory 1/12, 119234 Moscow, Russia; styx@biophys.msu.ru (S.S.K.); fursova@biophys.msu.ru (P.V.F.); solovchenko@mail.bio.msu.ru (A.E.S.); riznich46@mail.ru (G.Y.R.); rubin@biophys.msu.ru (A.B.R.); 2 Institute of Natural Sciences, Derzhavin Tambov State University, Internatsionalnaya Str. 33, 392000 Tambov, Russia; 3 Laboratory of Integrated Environmental Research, Pskov State University, Lenin Sq. 2, 180000 Pskov, Russia; taras_an@mail.ru

**Keywords:** polyphosphate accumulation, phosphate starvation, luxury uptake, mathematical model, regulation of phosphate metabolism

## Abstract

Using a mathematical simulation approach, we studied the dynamics of the green microalga *Chlorella vulgaris* phosphate metabolism response to shortage and subsequent replenishing of inorganic phosphate in the medium. A three-pool interaction model was used to describe the phosphate uptake from the medium, its incorporation into the cell organic compounds, its storage in the form of polyphosphates, and culture growth. The model comprises a system of ordinary differential equations. The distribution of phosphorous between cell pools was examined for three different stages of the experiment: growth in phosphate-rich medium, incubation in phosphate-free medium, and phosphate addition to the phosphorus-starving culture. Mathematical modeling offers two possible scenarios for the appearance of the peak of polyphosphates (*PolyP*). The first scenario explains the accumulation of *PolyP* by activation of the processes of its synthesis, and the decline in PolyP is due to its redistribution between dividing cells during growth. The second scenario includes a hysteretic mechanism for the regulation of PolyP hydrolysis, depending on the intracellular content of inorganic phosphate. The new model of the dynamics of P pools in the cell allows one to better understand the phenomena taking place during P starvation and re-feeding of the P-starved microalgal cultures with inorganic phosphate such as transient PolyP accumulation. Biotechnological implications of the observed dynamics of the polyphosphate pool of the microalgal cell are considered. An approach enhancing the microalgae-based wastewater treatment method based on these scenarios is proposed.

## 1. Introduction

In nature, microalgae frequently encounter the stress caused by the lack of mineral nutrients such as nitrogen, phosphorus (P), and sulfur, the key constituents of amino acids, proteins, lipids, photosynthetic pigments, vitamins, and other vital compounds. This stress profoundly affects the physiological condition of the microalgal cells. During their evolution, microalgae have developed various adaptations for coping with the nutrient depletion in their natural habitats. Studies of these mechanisms are relevant not only for understanding the nature of the stress action per se but also for microalgal biotechnology since microalgal cells respond to mineral nutrient starvation by accumulating valuable products such as carbohydrates and lipids [1,2] or producing hydrogen [3,4]. It is well known that P-starving cells of microalgae and cyanobacteria accumulate polyphosphates (*PolyP*) at an increased rate after replenishing phosphate in their growth medium [5,6,7]. This metabolic feature elicited a keen interest of researchers for many years since it opens new possibilities for wastewater treatment with microalgae, on the one hand, and using the resulting algal biomass enriched with P as a fertilizer, on the other hand [8,9].

Several models have been proposed that describe the dynamics of *PolyP* accumulation after a P famine period [10,11,12,13]. These models are formulated as a set of differential equations and originate from Droop’s model [14], describing the flow of mineral substances into the cell, their accumulation in the form of the cellular pool, and the corresponding kinetics of growth of the culture. The capacity or size of the cellular pool of the accumulated nutrient was limited by the maximum cell quota, that is, the maximum possible content of this mineral in the cell. John and Flynn [11] proposed to consider the accumulation of phosphate in various cell pools using the three-compartment phosphate interaction model (PIM). The PIM considers a pool of *PolyP*, a pool of soluble inorganic phosphate (*P_i_*), and a pool of phosphometabolites organic (*P_o_*) incorporated into structural components of the cell. The growth of culture was described by an exponential equation. John and Flynn [11] compared the description of PolyP accumulation by a model containing the three pools with that obtained from a model based on a single common pool of P. Qualitatively, the dynamics of phosphorus accumulation predicted by both models were similar, but the authors emphasize that separating the processes of phosphate assimilation and transport (this is important for organisms living under fluctuating phosphate availability) requires the use of a three-component model. The three-component model was extended (exPIM) by a function taking into account the effect of the competition of cells for light on the growth rate [10]. However, the simulation with the exPIM model did not reveal new qualitatively distinct regimes compared to PIM.

The kinetics of polyphosphate content can have various patterns and is determined by both the phosphate content in the medium and the internal state of the cell. Under ample nutrient conditions, *PolyP* content in the growing cell culture first decreases and begins to increase only after about two days [15], while the opposite pattern is observed after replenishing of phosphate in the medium after a period of starvation: the maximum of *PolyP* is quickly reached followed by its consumption [6,15]. This indicates that the cell can implement various mechanisms for regulating the accumulation of *PolyP*, leading to different kinetic patterns in the pool of *PolyP*, and an important task of mathematical modeling is to identify such mechanisms.

The aim of this work was to build a model that would describe the different modes of phosphate uptake and *PolyP* accumulation under conditions of sufficient nutrient supply or after phosphate starvation followed by phosphate replenishment. Testing and calibrating a mathematical model requires experimental data sets that reflect the kinetics of model variables under the various conditions described above. For modeling purposes, it was important for us to find experimental data for different patterns of accumulation and consumption of *PolyP*; we found such data in a seminal work on *Chlorella* by Aitchison and Butt [15]. Using this model, we tried to elucidate the mechanisms that determine the direction of change (increase or decrease) in the pool of cell *PolyP* and identify culture growth phase(s) with a maximum accumulation of *PolyP*.

To the best of our knowledge, this is the first attempt to find the common pattern of *PolyP* changes as a function of phosphate availability not only in the cultures grown in batch mode but also in (semi-)continuous cultures. These findings would be highly relevant for environmental biotechnologies for efficient biocapture of P from waste streams with microalgal cultures. Of separate importance is a comprehensive nature of the developed modeling approach allowing for a better understanding of the non-intuitive phenomena taking place during P starvation and subsequent re-feeding of the P-starved cells, e.g., the transient *PolyP* accumulation after the re-feeding.

## 2. Materials and Methods

### 2.1. Differential Equations for the 3-Pool Model of Phosphate Uptake

To describe the accumulation and use of phosphate and related compounds and the formation of P pools, the system of ordinary differential equations (ODE) was used. For the numerical solution of the ODE system and identification of the parameters, we used the DBSolve software package [16].

### 2.2. Model Calibration Using Experimental Data

Experimental data from the work by Aitchison and Butt [15] were taken to calibrate the model. In the cited paper, a three-stage experiment was performed. During stage I, *Chlorella vulgaris* was cultivated for 5 days (120 h) in a medium with excess phosphate content (3.23 mM). According to [15], by this time, cell division had fallen to a slow linear rate and then remained constant for several days (stationary growth phase). Aitchison and Butt [15] have shown that the cessation of growth was not due to the exhaustion of phosphate or any other nutrient in the medium. At the end of stage I, cells were harvested by centrifugation, resuspended in P-free medium, and incubated for 36 h (stage II). At the end of stage II, cells were harvested by centrifugation, resuspended in a medium with ample phosphate content (3.23 mM), and incubated for 24 h. Cell density and per-cell content of various P-containing fractions (total phosphate, orthophosphate, acid-soluble and acid-insoluble polyphosphates, lipid phosphate, KOH-soluble phosphate, trichloroacetic acid-soluble phosphate, phosphate in residue after alkaline extraction) were measured during all three stages of the experiment (see [15] for details).

Numeric data about cell density and polyphosphate content in cells during the first phase of the experiment were taken from Figure 2 in [15], acid-soluble and acid-insoluble fractions of polyphosphate were combined into a single *PolyP* pool. In a similar fashion, data on orthophosphate (i.e., inorganic phosphate *P_i_*) and polyphosphate (*PolyP*) content during stages II and III of the experiment were taken from Figures 3 and 4 in [15]. In particular, *PolyP* per-cell content was taken as a sum of □ and ■ from Figure 2 in [15] for stage I of the experiment, as a sum of △ and ▲ from Figure 3 in [15] for stage II, and as a sum of △ and ▲ from Figure 4 in [15] for stage III. Intracellular inorganic phosphate content during P starvation was taken as ⊙ from Figure 3 in [15]. The content of organic P-containing compounds *P_o_* was estimated by subtracting orthophosphate and polyphosphate content from the total phosphate content: □ minus the sum of △, ▲, and ⊙ from Figures 3 and 4 in [15] for stages II and III, respectively.

## 3. Results

### 3.1. Construction of the 3-Pool Model of P Species Interconversions (Version 1)

First, to describe the experimental kinetics, we took the existing PIM [11] and exPIM [10] models, in which PolyP is synthesized from inorganic phosphate. However, using such models, it was possible to describe only the increasing dynamics of PolyP; but attempts to describe the decrease in *PolyP* content under conditions with an excess of phosphate were unsuccessful. Since it is known that ATP and other phosphorylated molecules are involved in the synthesis and hydrolysis of PolyP, we assumed that phosphate fluxes in the cell should be described differently, namely, that the PolyP is synthesized from organic P-containing compounds. In addition, we assumed that the size of the minimum pool quotas can also play a role in the dynamics observed in the experiment and included the corresponding minimum quotas into all equations.

**Figure 1:**
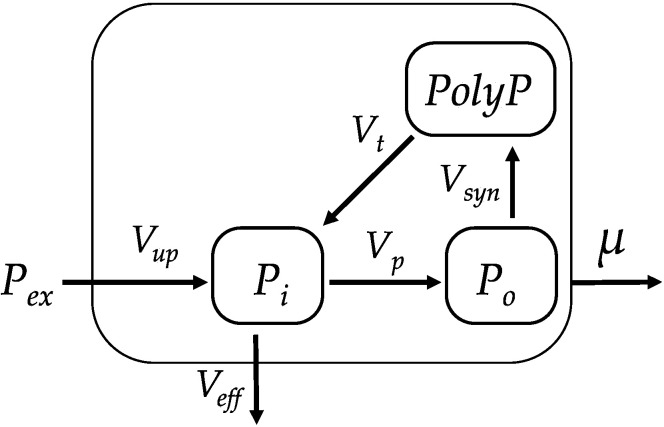
Pathways of the conversion of P in the algae cell. *P_ex_*—inorganic phosphate in the medium, *P_o_*—phosphometabolites (organic P-containing compounds), *P_i_*—inorganic phosphate, *PolyP*—polyphosphate. *V_up_* is the rate of phosphate uptake by the cell, *μ* is the specific growth rate of the culture, *V_p_* is the rate of formation of organic compounds from *P_i_*, *V_syn_* is the rate of *PolyP* synthesis, *V_t_* is the rate of *PolyP* hydrolysis, and *V_eff_* is the rate of *P_i_* efflux from the cell.

The structure of the model is presented in Figure 1. Symbol definitions and units are given in Table 1. Inorganic phosphate from the medium (*P_ex_*) enters the cell at a rate of *V_up,_* replenishing the intracellular inorganic pool (*P_i_*). *P_i_* from that pool either gets incorporated into organic phosphometabolites (ATP, DNA, RNA, phospholipids, etc.) denoted as (*P_o_*) at a rate of *V_p_* or flows back to the medium at a rate of *V_eff_*. Part of the *P_o_*, primarily in the form of ATP, at a rate *V_syn_* is converted into polyphosphates (*PolyP*), which is a polymer from phosphoric acid residues (n·PO^3−^_4_). *PolyP* can then decompose at a rate *V_t_* to *P_i_*. Cell division occurring at a rate *μ* depletes all cell P pools (Figure 1). Corresponding equations are presented in Table 2.

Growth of the culture (1) is described by the equation of logistic growth, which slows down over time with an increase in cell density even when all nutrients are ample, and the culture reaches the steady state. Here $\mu =\mu \prime \left(P_{o}\right)\cdot \left(1-\frac{C}{K_{f}}\right)$ is the specific culture growth rate (1/*h*), which decreases linearly with an increase in cell density and depends on the cell content of organic *P_o_* species (see below). *K_f_* is the medium capacity (maximum number of cells in the medium, which is reached at stationary growth phase). Such limitation is typical for experiments with periodic culture growth and is usually explained by self-shading of cells in a dense culture, which makes light a limiting factor for culture growth [10].

Equation (2) describes the phosphate uptake from the medium. Equation (3) describes the change in the intracellular *P_i_* pool: it increases due to the phosphate uptake from the medium and *PolyP* hydrolysis and decreases due to *P_i_* incorporation into organic compounds, efflux from the cell, or due to formation of new cells (cell division). Equations (4) and (5) describe, respectively, changes in the pools of *P_o_* and *PolyP*.

To obtain a specific form of expressions on the right side of the equations, we introduced the parameters taking into account the maximum and minimum cell quota size for P pools: $Q_{P_{o}\max}$, $Q_{P_{i}\max}$, and $Q_{PolyP\max}$ are the maximum quota and $Q_{P_{o}\min}$, $Q_{P_{i}\min}$, and $Q_{PolyP\min}$ are the corresponding minimum quota sizes for intracellular *P_o_*, *P_i_*, and *PolyP* pools, respectively. The maximum quota does not let each pool be overfilled: the reactions filling the pool decelerate when the pool gets close to its maximum quota. Similarly, the minimum quota does not let each pool be underfilled: the reactions consuming the pool decelerate when the pool gets close to its minimum quota. Most reaction rates follow Michaelis–Menten-like kinetics, which can be characterized by half-saturation (Michaelis) constants *K*: reaction rate reaches half of its maximum value when its substrate pool content goes beyond its minimum quota by *K*.

We assume that the external phosphate uptake occurs in accordance with the Michaelis–Menten kinetics and is limited by the maximum quota for intracellular *P_i_*, $Q_{P_{i}\max}$. As suggested above, *µ* decreases with an increase in cell density. We also assume that *µ* is limited by the minimum cell quota of organic P-containing compounds $Q_{P_{o}\min}$: cell division stops when their content reaches $Q_{P_{o}\min}$. Finally, the expressions for the corresponding rates assume the forms shown in Table 3.

### 3.2. Model Validation

**Figure 2:**
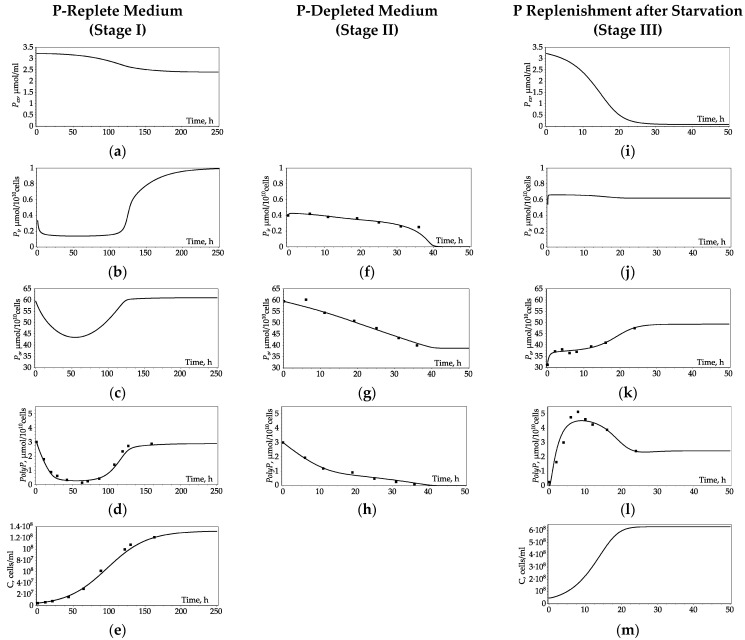
Dynamics of P pools and cell density during *Chlorella* culture growth in P-replete medium (**a**–**e**), P-depleted medium (**f**–**h**), and when an excess of P is added to the medium after a period of depletion (**i**–**m**). Dots—experimental data from [15] (refer to Section 2.2 for details), solid lines—simulation results. (**a**,**i**)—External phosphate (*P_ex_*), (**b**,**f**,**j**)—Per-cell *P_i_* content, (**c**,**g**,**k**)—per-cell *P_o_* content, (**d**,**h**,**l**)—per-cell *PolyP* content, (**e**,**m**)—cell density. The underlying experimental data are from [15]: (**d**)—sum of □ and ■ from Figure 2 in [15], (**e**)—● from Figure 2 in [15], (**f**)—⊙ from Figure 3 in [15], (**g**)—□ minus sum of △, ▲, and ⊙ from Figure 3 in [15], (**h**)—sum of △ and ▲ from Figure 3 in [15], (**k**)—□ minus sum of △, ▲, and ⊙ from Figure 4 in [15], (**l**)—sum of △ and ▲ from Figure 4 in [15].

As mentioned above, experimental data presented in [15] (dots on the graphs, Figure 2) were taken to verify the model. Three experimental stages were considered: cultivation in a medium with excess phosphate (stage I), incubation in P-free medium (stage II), and phosphate replenishment after starvation (stage III). We hypothesize that phosphorus depletion is causing significant changes in cell metabolism, so we used different sets of parameters for each stage of the experiment. These parameters were obtained by fitting the model to the experimental data (Supplementary Materials). Simulated kinetics of changes in the P pools was obtained for each stage (Figure 2).

#### 3.2.1. Culture Growth under Ample External Phosphate Conditions (Stage I)

Stage 1 of the experiment [15] is started by inoculation of fresh medium with *Chlorella* cells taken from the stationary-phase culture featuring a high *PolyP* content. The experimental curves for cell density and intracellular *PolyP* content obtained during culture growth in P-replete medium were fitted using the model (Figure 2d,e). For the derived set of the model parameters, the dynamics of the remaining variables of the model (external phosphate *P_ex_* and per-cell content of *P_i_* and *P_o_*) were obtained (Figure 2a,b,c).

The culture growth starts without a lag phase, but until the 50th hour of cultivation, all three intracellular P pools (*P_o_*, *P_i_*, and *PolyP*) decrease. After the 50th hour, *P_o_* begins to increase, and by the 110th hour, it reaches saturation. The *PolyP* pool also increases and reaches saturation, but with a time lag of about 10 h. By the 110th hour, the culture growth slows down, the culture enters the stationary growth phase, and the intracellular *P_i_* pool begins to increase sharply. After 200 h of cultivation, when the growth rate of the culture significantly decreases and reaches a plateau, *P_ex_* decreases almost one and a half times to its initial value, phosphate uptake rate turns negligible, and the intracellular P pools reach their steady-state levels.

#### 3.2.2. Phosphate Deficiency (Stage II)

In this stage of the experiment, the algae were transferred into a P-free medium [15]. Here we assumed that both *V_up_* = 0, and *μ* = 0. We took model variable values at the 120th hour of stage I as the initial values for stage II. The experimental curves of intracellular *P_i_*, *P_o_*, and *PolyP* content were fitted using the model (Figure 2f–h). As one can see from the experimental and model curves, during this stage, all P pools are depleted to their minimum values in 40 h due to *P_i_* efflux to the medium.

#### 3.2.3. Phosphate Replenishment after Starvation (Stage III)

After starvation, cells were resuspended in the P-rich medium with phosphate content as in stage I [15]. The experimental curves of intracellular *P_o_* and *PolyP* content were fitted using the model (Figure 2k,l). For the derived set of the model parameters, the dynamics of the remaining variables of the model (external phosphate *P_ex_*, per-cell content of *P_i_*, and cell density *C*) were obtained (Figure 2i,j,m).

As a result of phosphate replenishment, rather rapid changes of P pools are observed within the first 24 h. During this time, almost all external phosphate, *P_ex,_* is absorbed from the medium (Figure 2i). Culture growth resumes with the lag phase first and then reaches its steady state (Figure 2m). The pool of *P_i_* increases rather rapidly from 0.54 to 0.76 μmol 10^−10^cells^−1^, then begins to decrease slightly (Figure 2j). The pool of *PolyP* in the first 10 h increases almost two times compared to its value before starvation, but during the next 10 h, it decreases again (Figure 2l). The pool of *P_o_* also increases during this period and then remains at a stationary level (Figure 2k).

### 3.3. The Model Update Taking into Account the Regulation of Phosphate Metabolism (Version 2)

Analyzing the model kinetics of phosphate pools and cell growth after adding phosphate to a starving culture, we found that it is possible to describe the increase and subsequent decrease in the pool of *PolyP* only if the culture begins to grow intensely, and as a result, external phosphate is depleted. However, Aitchison and Butt indicated that the external phosphate was in excess, and the growth of the culture was negligible [15]. To explain the occurrence of the *PolyP* peak under such conditions, we assumed that there is a mechanism for regulating the activity of enzymes involved in phosphorus metabolism. According to proteomics data regarding the expression of the Vtc complex, which is involved in the synthesis of *PolyP* [6], we assumed that the key stage of regulation might be the synthesis of *PolyP*. However, all our attempts to find a set of parameters for obtaining the kinetics corresponding to the scenario without culture growth were unsuccessful. So, we assumed that the key stage might be the hydrolysis of the *PolyP* pool regulated by some regulator *R*, which depends on the *P_i_* level in the cell. Based on the observed dynamics of the *PolyP* pool, we assumed that *R* activates hydrolysis of *PolyP*. *R* reaches its maximum under ample external phosphate, and consequently, the rate of *PolyP* hydrolysis can also reach its maximum. During starvation, the *P_i_* level decreases, and the *R* level also decreases, blocking the hydrolysis of the *PolyP* pool. Phosphate replenishment after starvation causes a slow rise in the *R* level, thereby unlocking *PolyP* hydrolysis.

To describe the dynamics of *R*, we use the equation

$$\frac{dR}{dt}=\frac{V_{R}\cdot {\left(P_{i}-Q_{P_{i}\min}\right)}^{n}}{{\left(P_{i}-Q_{P_{i}\min}\right)}^{n}+K_{R}^{n}}\frac{Q_{R\max}-R}{Q_{R\max}}-k\cdot R,$$

in which the formation of *R* depends on cooperative binding of *P_i_* in Hill–Langmuir form with Hill coefficient *n*, and takes into account the *P_i_* minimum quota, *Q_*min*Pi_*, and the regulator *R* maximum quota, *Q_*max*R_*. The decay of the regulator is proportional to its concentration with the rate constant *k*. We assumed that the rate of hydrolysis of *PolyP* is proportional to *R*/*Q_R*max*_*; thus, the expression (10) for *V_t_* is transformed into the following:

$$V_{t}=\frac{V_{6}\cdot (PolyP-Q_{PolyP\min})}{PolyP-Q_{PolyP\min}+K_{6}}\cdot \frac{Q_{P_{i}\max}-P_{i}}{Q_{P_{i}\max}}\cdot R/Q_{R\max}.$$

**Figure 3:**
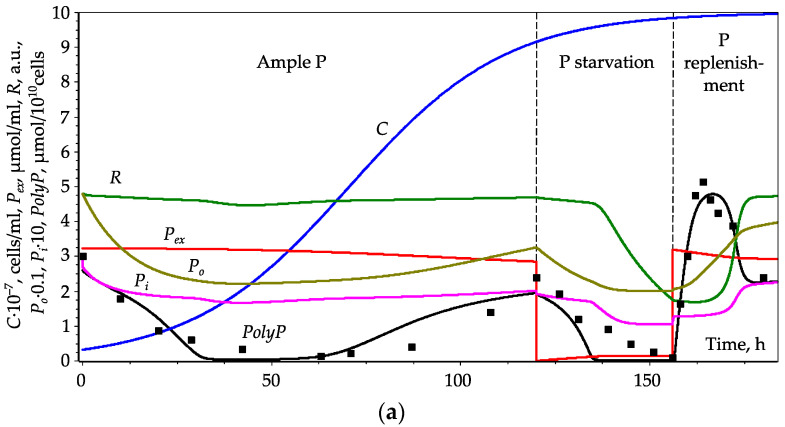

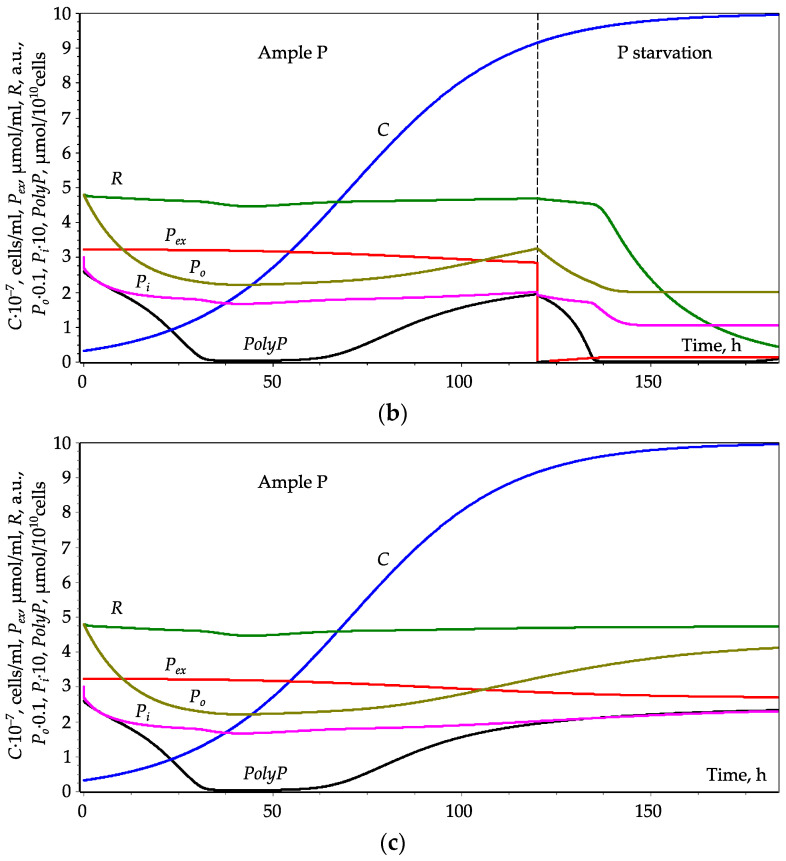
Dynamics of P pools, regulator content, and cell density during *Chlorella* culture growth in three experimental stages. (**a**)—Three-stage experiment where cells were grown in P-replete medium for 120 h, then transferred to P-depleted medium, and after 36 h of starvation, an excess of phosphate was added to the medium. (**b**)—No addition of external phosphate after starvation. (**c**)—No phosphate depletion. Dots—experimental data for *PolyP* from [15] (refer to Section 2.2 for details)—sum of □ and ■ from Figure 2 in [15], and sum of △ and ▲ from Figures 3 and 4 in [15] with corresponding time shifts (120 and 156 h, respectively). Solid lines—simulation results: red—external phosphate (*P_ex_*), magenta—per-cell *P_i_* content (*P_i_*·10), brown—per-cell *P_o_* content (*P_o_*·0.1), black—per-cell *PolyP* content, green—regulator content (*R*·100), blue—cell density (*C*·10^−7^).

As a result of fitting the model to experimental data, we managed to obtain a unified set of parameters for which the dynamics of P pools for different stages of the experiment are adequately described (Figure 3a). The dynamics of P pools at stage I are close to that for model version 1 (Section 3.2.1). This is due to the fact that since external phosphate is in excess, the level of the *R* is therefore almost constant and does not affect the dynamics of other variables.

After 120 h of culture growth, when it is transferred to P-free medium (*P_ex_* = 0, stage II of the experiment), a gradual decrease in *PolyP* occurs as a result of its hydrolysis. During this stage, the level of *P_i_* decreases, and, consequently, the level of the regulator *R* decreases. A low concentration of *R* leads to a decrease in the hydrolysis of *PolyP*. As a result, at the next stage III, in response to the addition of *P_ex,_* a rapid accumulation of *PolyP* occurs. Other P pools also increase. An increase in *P_i_* causes an increase in *R*, which finally leads to the hydrolysis of the over-synthesized *PolyP* delayed by several hours after P replenishment. Thus, *PolyP* shows a distinct peak followed by a decline to steady-state level.

Without the addition of external phosphate *P_ex_*, the blockade of *PolyP* hydrolysis does not lead to its accumulation since the pool of *P_o_* is depleted (Figure 3b). If *P_ex_* is ample throughout the experiment, no *PolyP* peak is observed during culture growth (Figure 3c), and the steady-state level of *PolyP* is close to that at the end of stage III (Figure 3a).

### 3.4. Effect of Duration of Phosphate Starvation

**Figure 4:**
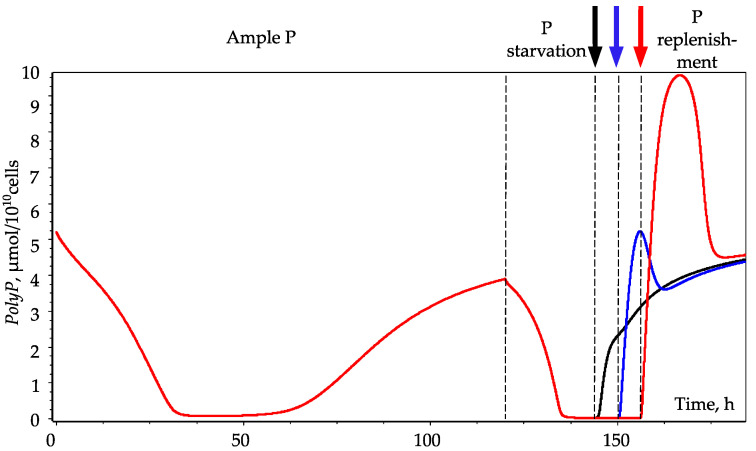
Dynamics of *PolyP* pool during *Chlorella* culture growth in three-stage experiment. Cells were grown in P-replete medium for 120 h and then transferred to P-depleted medium (dashed line). An excess of phosphate was added to the medium after 24 h (black curve and arrow), 30 h (blue curve and arrow), or 36 h (red curve and arrow) of depletion.

It was indicated by Aitchison and Butt [15] that the duration of P starvation affects the magnitude of the *PolyP* peak. In model experiments, we found that if the duration of P starvation is reduced from 36 to 30 h, the *PolyP* peak significantly decreases. When the duration decreases to 20 h, the peak disappears, which is in qualitative agreement with [15] (Figure 4).

**Figure 5:**
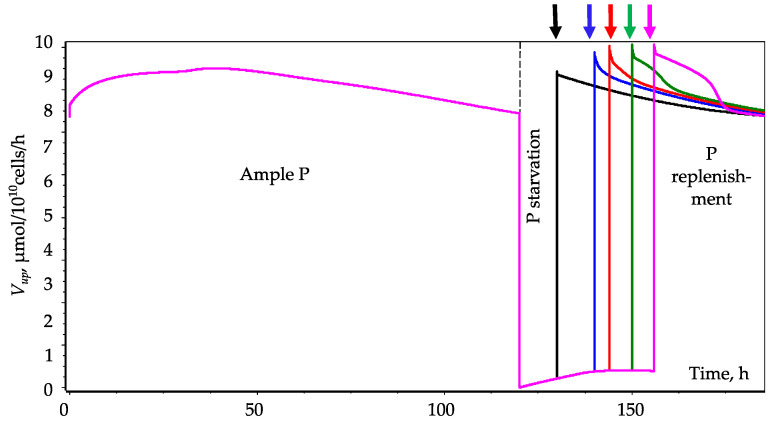
Dynamics of phosphate uptake rate *V_up_* during *Chlorella* culture growth in three-stage experiment. Cells were grown in P-replete medium for 120 h and then transferred to P-depleted medium (dashed line). An excess of phosphate was added to the medium after 5 h (black curve and arrow), 20 h (blue curve and arrow), 24 h (red curve and arrow), 30 h (green curve and arrow), or 36 h (magenta curve and arrow) of depletion.

In the cited paper [15], it was also shown that with an increase in the duration of starvation, the phosphate uptake rate *V_up_* also increases. The dynamics of *V_up_* were modeled for different durations of starvation (Figure 5). An increase in the duration from 0 to 20 h leads to a correspondent increase in the maximum uptake rate *V_up_*; a further increase in the duration of starvation does not change the maximum value of *V_up_*.

### 3.5. Conditions for the Maximum PolyP Yield

**Figure 6:**
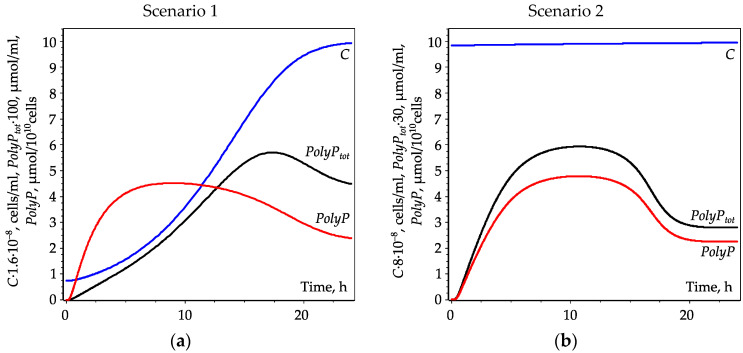
Dynamics of *PolyP* and *PolyP_tot_* in microalgal cell culture. Red curves—per-cell polyphosphate content (*PolyP*), black curves—total polyphosphate content (*PolyP_tot_*), blue curves—cell density (*C*). (**a**)—Scenario 1, intensely growing culture and depleting *P_ex_*. (**b**)—Scenario 2, stationary-phase culture and ample *P_ex_*.

For biotechnology, it is important to find the conditions when the maximum *PolyP* yield in the culture (*PolyP_tot_*) is reached. One can calculate the amount of *PolyP_tot_* in the culture as the product of the cell density and per-cell *PolyP* content: *PolyP_tot_* = *C*·*PolyP*. Two versions of the model allow describing two different scenarios for *PolyP* accumulation (Figure 6).

Scenario 1 describes an intensive growth of a cell culture leading to depletion of external phosphate *P_ex_*. In this scenario, *PolyP* and *PolyP_tot_* reach their maximum values at different moments of time: in approximately 8 and 16 h after phosphate replenishment, respectively, while the culture approaches its steady-state density at about the 30th h after phosphate replenishment (Figure 6a). Scenario 2 describes stationary-phase culture with a low division rate, so the cell density remains almost constant, and the external phosphate *P_ex_* remains ample. In this scenario, *PolyP* and *PolyP_tot_* reach their maximum values simultaneously: in approximately 8 h after phosphate replenishment (Figure 6b).

## 4. Discussion

Despite the keen interest in the problem of the accumulation of polyphosphates in algal cells, the exact mechanism of regulation of this process is still unclear. The accumulation of *PolyP* after phosphate addition to the starving culture can be guided by various scenarios. In this paper, we have considered two scenarios for its accumulation. In the first one, phosphate is added to the culture, which growth has been declined due to P starvation, as in [6], and then the culture begins to grow rather intensely and reaches the stationary phase in a short time. During this time, external phosphate is almost completely consumed, and the slowdown in cell growth is accompanied by a PolyP peak (scenario 1). Alternatively, phosphate is added to the culture at a stationary growth phase [15], the concentration of external phosphate varies only slightly, and the number of cells increases slightly; however, the peak of PolyP in cells is also observed (scenario 2). We have proposed two versions of the model in which the two scenarios of *PolyP* accumulation described above are realized. According to these versions, we can propose mechanisms of *PolyP* accumulation. We assume that the regulation of *PolyP* accumulation in each version is carried out in different ways. In addition, by modeling, we can clarify the external conditions that determine the dynamics of PolyP in a non-starving culture.

### 4.1. PolyP Accumulation without Prior Starvation (Stage I)

As shown in experiments, no *PolyP* accumulation occurs in an actively growing culture [15]. All phosphate absorbed by the cell is spent on the synthesis of *P_o_*, which is needed for the growth of the culture. If, in the experiment, cells that are in the stationary growth phase and have a *PolyP* reserve are used as an inoculum, then this reserve is spent on cell growth during the first days of cultivation.

The accumulation of *PolyP* begins when cell growth is limited, for example, due to their self-shadowing, while external phosphate is in excess. As the growth of the culture slows down, the amount of *PolyP* in the cells increases. As a result, when the growth of the culture reaches the stationary phase, the cell accumulates a certain amount of *PolyP*

### 4.2. PolyP Accumulation after P Replenish to Starved Culture (Stage III)

#### 4.2.1. P Addition Activates Culture Growth, and Whereby Is Depleted (Scenario 1)

In scenario 1, we did not explicitly introduce any regulation into the model. We obtained differences in the kinetics of *PolyP* due to different sets of values of the reaction rate parameters for different stages of the experiment. Here, implicit regulation is provided by a change in the reaction parameters. The Michaelis constant in the *PolyP* rate synthesis at stage III is two orders of magnitude less compared to its value for stage I, which corresponds to a significant increase in the rate of *PolyP* synthesis and explains the differences in the kinetics of *PolyP* at stages I and III at the same level of external phosphate (Figure 2). We may guess that the regulatory mechanism in scenario 1 is associated with Vtc complex expression. As it was shown in [6], P starvation is accompanied by the expression of the Vtc complex that may lead to the following activation of *PolyP* synthesis. After an increase in the level of *P_i_* in the cell, the expression of Vtc decreases, which leads to a decrease in *PolyP* synthesis; already synthesized *PolyP* gets distributed between the daughter cells in the process of cell division, and per-cell *PolyP* content decreases.

#### 4.2.2. P Is Added to the Culture at Stationary Growth Phase and Is Not Depleted (Scenario 2)

However, to describe scenario 2, it is not enough to change the model parameters. The introduction of the regulator *R*, which is sensitive to the level of phosphate *P_i_* in the cell, helped us to solve this problem. From the analysis of the experimental kinetics of phosphate pools in the cell [15], we guessed that the regulator primarily affects the hydrolysis of *PolyP*, regulating the degradation of *PolyP*. In sustained ample phosphate conditions (without starvation), the content of phosphate in the cell pools is in dynamic equilibrium (*P_i_* goes into *P*, *P* goes into *PolyP*, *PolyP* goes back to *P_i_*). When *P_i_* decreases as a result of starvation, the *R* level consequently decreases. This is the reason that after the addition of external phosphate, we do not observe equilibrium of these pools due to the rate of hydrolysis of *PolyP* (*V_t_*) is significantly reduced in comparison with the rate of its synthesis (*V_syn_*). Thus, the major part of the absorbed phosphate goes into the *PolyP* pool.

A comparison of the reaction rates *V_up_*, *V_syn,_* and *V_t_* shows that at the beginning of stage III, up to 80% of the absorbed external phosphate is stored in the form of *PolyP*. As a consequence, the *P_i_* level remains quite low for some time, although the level of external phosphate is high. Thus, even in the presence of an excess of external phosphate, the cell remains in a state of P deficiency, just like during starvation. The cell can leave this state only when the *PolyP* pool is saturated (approaches its maximum quota), which prevents the further synthesis of *PolyP*. After the *PolyP* pool is filled, the *P_o_* and *P_i_* pools begin to replenish. The regulator *R* begins to rise, which leads to an excess of the rate of hydrolysis (*V_t_*) over the rate of synthesis (*V_syn_*). The pool of *PolyP* begins to decrease, and phosphate metabolism returns to its original equilibrium.

We suppose that such a mechanism may be realized due to a positive feedback loop: a decrease in *P_i_* leads to a decrease in *R*, which results in a decrease in the rate of hydrolysis (*V_t_*) and a further decrease in *P_i_*. Thus, if *R* decreases below a certain critical level, the phosphate metabolism of the cell switches to an alternative mode, reorienting to the storage of *PolyP*. The phenomenon of hysteresis, i.e., the existence of two modes of phosphate metabolism under identical external conditions, is observed. The choice between these modes is determined by the backstory of the development of cell culture. This explains the dependence of the emergence of the *PolyP* peak on the duration of the fasting period: during short-term starvation, phosphorus metabolism does not have enough time to switch to the *PolyP* accumulation mode (Figure 4).

Regulation of almost all P-related enzymes by intracellular phosphate content has been studied for various organisms [17,18,19,20,21]. The most common way of this regulation involves SPX domains, which are present in several enzymes that promote the transport of phosphate across the outer and inner membranes of the cell, as well as in transcriptional regulators [22]. These SPX domains bind highly phosphorylated forms of inositol (inositol pyrophosphates IP_7_ and IP_8_), which are synthesized in the cell when phosphate is ample and hydrolyzed into forms with a smaller number of phosphate groups under phosphate deficiency [20,23]. Thus, we may assume inositol pyrophosphate to be a possible regulator involved in both described above mechanisms of the peak *PolyP* accumulation after P starvation.

We simulated two possible scenarios leading to excessive accumulation of *PolyP* in the cell. Although these scenarios are based on two different mechanisms of regulation of phosphorus metabolism, we assume that both mechanisms are implemented in the cell and act simultaneously. Their cumulative effect depends on the specific conditions of the experiment. Based on the available experimental data, we cannot be confident which of the effects, an increase in *PolyP* synthesis (scenario 1) or a decrease in *PolyP* hydrolysis (scenario 2), makes a greater contribution to its accumulation in a particular case. Similarly, we cannot say for sure which of the effects, the hydrolysis of *PolyP* (scenario 2) or the redistribution of *PolyP* between dividing cells during culture growth (scenario 1), contributes more to the decrease in *PolyP* after the peak.

## 5. Concluding Remarks: Biotechnological Implications

For biotechnology purposes, it is important to understand when a cell culture accumulates the maximum amount of *PolyP* and/or displays the maximum capacity for phosphate uptake. One might expect that the top *PolyP* accumulation is associated either with the moment of reaching the peak of *PolyP* or with reaching a stationary state by the culture. We have compared these two scenarios of accumulation of PolyP (Figure 6).

For scenario 1 (a culture growing after replenishing of external phosphate), the rate of *PolyP* accumulation by the culture peaks later (about 16 h after *P_ex_* replenishment) than the maximum of *PolyP* in the cell is reached (about 8 h after *P_ex_* replenishment), but before the onset of stationary phase (about 30 h after *P_ex_* replenishment). An important outcome of this work is that it allows understanding the phenomena taking place during the transition of microalgal cells from P starvation to ample P conditions. This understanding lays a foundation for knowledge-based solving of assorted biotechnological and environmental problems. Thus, to obtain biomass with the maximum *PolyP* content in scenario 1, one needs to harvest the biomass when cell division in the culture begins to slow down. If P removal from wastewater (“polishing”) is the goal, then the culture should be allowed to grow to the stationary phase (Figure 6a).

For scenario 2 (replenishing of external phosphate after starvation during early stationary), the culture and per-cell *PolyP* content peak simultaneously (ca. 8 h of growth after *P_ex_* replenishment). An important observation and conclusion based on the modeling is that a stationary-phase culture pre-starved of P accumulates *PolyP* faster than a rapidly growing exponential-phase culture. However, such a culture cannot be used for wastewater “polishing” since it does not take down the external phosphate; it is rather suitable for the removal of bulk P from the wastewater. Notably, in growing cultures (scenario 1), the maximum *PolyP* is reached when the level of phosphate in the medium is still high (Figure 6b).

Having said this, one may think that the goals of harvesting biomass enriched in *PolyP* and efficient wastewater treatment are mutually exclusive. This apparent limitation can be circumvented by the application of the two-stage cultivation approach common in microalgal biotechnology to bioremoval of P from wastewater. At the first stage, a batch of wastewater is amply inoculated with algal cells pre-starved of P (obtained, e.g., from a previous treatment cycle) to the density comparable to that of stationary-phase culture. Notably, the algal starvation in a P-free medium should be sufficiently long to deplete the cell P quota (about 36 h in case of [15]). When *PolyP* level peaks (about 8 h in the case studied here), most of the biomass is harvested for use as P biofertilizers.

The remaining cells divide until depletion of P in the current wastewater batch (the second stage). The proportion of harvested cell culture can be calculated from the phosphate content of the treated wastewater batch and cell density of the stationary-phase culture. After depletion of P in the treated batch, most of the treated wastewater is discharged after microalgal cell harvesting, and the remaining culture is incubated in the absence of P to produce the P-starved inoculum for the next batch of wastewater.

Overall, the findings outlined above take us closer to a more comprehensive understanding of patterns governing the (i) phosphate uptake from the medium and (ii) turnover of PolyP reserves in the cell. Another valuable outcome of the modeling effort is that it enables biotechnologists to make knowledge-based decisions about the design and potential efficiency of bioprocesses ensuring sustainable sequestration of P with both batch and (semi)continuous algal cultures. Finally, the decisions on the production culture management in microalgal biotechnology would be made with more confidence when they are backed by the analysis employing a more comprehensive model of algal P nutrition.

## Figures and Tables

**Table 1:** Symbol definitions and units.

Symbol	Unit	Defenition
*P_ex_*	μmol/mL	Inorganic phosphate in the medium
*P_i_*	μmol/10^10^cells	Inorganic phosphate in cells
*P_o_*	μmol/10^10^cells	Phosphometabolites in cells
*PolyP*	μmol/10^10^cells	Polyphosphates in cells
*C*	Cells/mL	Cell density
*R*	Arbitrary	Regulator in cells
μ,μ’	1/h	Specific growth rate
*V_up_*	μmol/(10^10^cells∙h)	Inorganic phosphate uptake rate
*V_eff_*	μmol/(10^10^cells∙h)	Inorganic phosphate efflux rate
*V_p_*	μmol/(10^10^cells∙h)	Rate of phosphometabolites synthesis
*V_syn_*	μmol/(10^10^cells∙h)	Rate of polyphosphates synthesis
*V_t_*	μmol/(10^10^cells∙h)	Rate of polyphosphates hydrolysis

**Table 2:** Equations describing processes in Figure 1.

Process	Equation	Units	
Cell division rate	$\frac{dC}{dt}=\mu^{\prime }\cdot (1-\frac{C}{K_{f}})\cdot C=\mu \cdot C$	$\frac{\mathrm{cells}}{\mathrm{mL}\cdot h}$	(1)
External phosphate *P_ex_* uptake by cells	$\frac{dP_{ex}}{dt}=-V_{up}\cdot C\cdot 10^{-10}+V_{eff}\cdot C\cdot 10^{-10}$	$\frac{\mathrm{µmol}}{\mathrm{mL}\cdot h}$	(2)
Intracellular *P_i_* pool change	$\frac{dP_{i}}{dt}=V_{up}-V_{p}+V_{t}-V_{eff}-\mu \cdot P_{i}$	$\frac{\mathrm{µmol}}{10^{10}\;\mathrm{cells}\cdot h}$	(3)
Intracellular *P_o_* pool change	$\frac{dP_{o}}{dt}=V_{p}-V_{syn}-\mu \cdot P_{o}$	$\frac{\mathrm{µmol}}{10^{10}\;\mathrm{cells}\cdot h}$	(4)
Intracellular *PolyP* pool change	$\frac{dPolyP}{dt}=V_{syn}-V_{t}-\mu \cdot PolyP$	$\frac{\mathrm{µmol}}{10^{10}\;\mathrm{cells}\cdot h}$	(5)

**Table 3:** Rate expressions. *V*_1,2…7_ are the maximum rates; *K*_1,2…7_ are the half-saturation constants (Michaelis constants); $Q_{P_{o}\max}$, $Q_{P_{i}\max}$, and $Q_{PolyP\max}$ are the maximum quotas for organic phosphorus-containing compounds, intracellular inorganic phosphate, and polyphosphate pools;$\;Q_{P_{o}\min}$, $Q_{P_{i}\min}$, and $Q_{PolyP\min}$ are the corresponding minimum quotas.

Process	Equation	Units	
*P_i_* uptake rate	$V_{up}=\frac{V_{1}\cdot P_{ex}^{}}{P_{ex}^{}+K_{1}}\cdot \frac{Q_{P_{i}\max}-P_{i}}{Q_{P_{i}\max}}$	$\frac{\mathrm{µmol}}{10^{10}\;\mathrm{cells}\cdot h}$	(6)
Specific growth rate of the culture	$\mu =\frac{V_{2}\cdot (P_{o}-Q_{P_{o}\min})}{P_{o}-Q_{P_{o}\min}+K_{2}}\cdot (1-\frac{C}{K_{f}})$	$\frac{1}{h}$	(7)
Conversion rate of intracellular *P_i_* to organic P-compounds	$V_{p}=\frac{V_{3}\cdot (P_{i}-Q_{P_{i}\min})}{P_{i}-Q_{P_{i}\min}+K_{3}}\cdot \frac{Q_{P_{o}\max}-P}{Q_{P_{o}\max}}$	$\frac{\mathrm{µmol}}{10^{10}\;\mathrm{cells}\cdot h}$	(8)
*PolyP* formation rate	$V_{syn}=\frac{V_{5}\cdot (P_{o}-Q_{P_{o}\min})}{P_{o}-Q_{P_{o}\min}+K_{5}}\cdot \frac{Q_{PolyP\max}-PolyP}{Q_{PolyP\max}}$	$\frac{\mathrm{µmol}}{10^{10}\;\mathrm{cells}\cdot h}$	(9)
*PolyP* hydrolysis rate	$V_{t}=\frac{V_{6}\cdot (PolyP-Q_{PolyP\min})}{PolyP-Q_{PolyP\min}+K_{6}}\cdot \frac{Q_{P_{i}\max}-P_{i}}{Q_{P_{i}\max}}$	$\frac{\mathrm{µmol}}{10^{10}\;\mathrm{cells}\cdot h}$	(10)
Rate of *P_i_* efflux	$V_{eff}=\frac{V_{7}\cdot (P_{i}-Q_{P_{i}\min})}{P_{i}-Q_{P_{i}\min}+K_{7}}$	$\frac{\mathrm{µmol}}{10^{10}\;\mathrm{cells}\cdot h}$	(11)

## Supplementary Materials

The following are available online at https://www.mdpi.com/article/10.3390/cells10123571/s1, Table S1: Initial values for model variables, Table S2: Parameters for model version 1, Table S3: Parameters for model version 2.
