# Supplementary material for: Simulating the Interplay between the Uptake of Inorganic Phosphate and the Cell Phosphate Metabolism under Phosphorus Feast and Famine Conditions in Chlorella vulgaris
Source: Cells. 2021 Dec 17;10(12):3571. doi: 10.3390/cells10123571 (PMC8700456; doi:10.3390/cells10123571)
Supplement: Supplementary file 1 [file cells-10-03571-s001.zip › cells-1488140-supplementary.pdf]

Supplementary Materials

Table S1. Initial values for model

variables	Ample P (stage I)	Starvation (stage II)	Replenishment (stage III)
C (cells/ml)	$4 \cdot 10^6$	$1.3 \cdot 10^8$	$1.3 \cdot 10^8$
P_{ex} (μmol/ml)	3.23	0	3.23
P_i (μmol/10^{10}cells)	0.4	0.34	0.54
P_o (μmol/10^{10}cells)	59.5	59.5	31.2
PolyP (μmol/10^{10}cells)	3	3	0.1

Table S2. Parameters for model version 1

	Ample P (stage I)	Starvation (stage II)	Replenishment (stage III)
K1	0.12		703.52
K2	0.00		780.11
K3	0.19	120.93	135.51
K5	123.50	1.81	1.04
K6	0.08	0.16	0.01
K7	0.01	0.01	0.01
Kf	1.32E+08		6.29E+08
V1	1.54		25267.31
V2	0.04		29.30
V3	100.04	404.85	5582.12
V5	7.89	1.99	5.68
V6	1.11	2.14	38.26
V7	100.02	0.62	0.27
Q_p	61.85		60.41
Q_pi	1.00		0.71
Q_pp	9.13		20.04
Qp	30.00	38.67	32.02

Table S3. Parameters for model version 2

	All stages
K1	74.84
K3	0.19
K5	1.69
K6	0.00
K7	0.52
Kf	1.25E+08
KmR	0.07
V1	44.00
V2	0.05
V3	81.51
V5	26.18
V6	22.23
V7	5.53
VR	0.05
kR	0.05
n	4
